# Exploring the association between social support and anxiety during major public emergencies: a meta-analysis of the COVID-19 pandemic

**DOI:** 10.3389/fpubh.2024.1344932

**Published:** 2024-07-09

**Authors:** Jianmei Liu, Siyu Chang, Zhidan Wang, Fasahat Z. Raja

**Affiliations:** ^1^School of Public Policy and Management, China University of Mining and Technology, Xuzhou, China; ^2^School of Education Science, Jiangsu Normal University, Xuzhou, China

**Keywords:** COVID-19, social support, anxiety, culture, pandemic phrase, major public emergencies

## Abstract

**Objective:**

The COVID-19 pandemic has prompted a surge in research focusing on mental health issues faced by society, with particular emphasis on the interplay between social support and anxiety. However, the results of these studies have often been controversial.

**Methods:**

To address this, we conducted a meta-analysis of 104 studies (*N* = 107,660) to investigate the relationship between anxiety and social support and the potential moderate variables.

**Results:**

Our meta-analysis revealed a negative correlation between social support and anxiety (*r* = −0.233). The study also demonstrated the variation in the relationship between social support and anxiety was moderated by cultural area (*Q* = 14.120, *p* < 0.05) and phrase of the pandemic (*Q* = 13.678, *p* < 0.05).

**Conclusion:**

The relationship between social support and anxiety can differ across different cultural areas and throughout the phrase of the pandemic. Consequently, we advocate for a nuanced assessment of the role of social support in mitigating public anxiety, taking into account the mediating effects of these factors in the context of major public emergencies.

## Introduction

The outbreak of the Coronavirus Disease 2019 (COVID-19) has had a profound and far-reaching impact on a global scale. The health implications of the virus have been severe, with millions of confirmed cases and over three million deaths reported globally as of now. The social and psychological impacts of the pandemic have also been significant. Social distancing measures and lockdowns have led to increased loneliness and isolation, particularly for vulnerable populations such as the older adult and those with pre-existing mental health conditions (1–3). The uncertainty and fear surrounding the virus have also led to a rise in anxiety and depression levels among the general public (4–6). According to the World Health Organization (WHO), the ongoing pandemic could trigger a significant increase in anxiety disorders by more than 25%, and the annual economic burden associated with anxiety and depressive disorders is projected to approximate $1 trillion (7). The widespread anxiety experienced during the pandemic underscores the urgency for empirical research to investigate effective strategies for managing anxiety during the major public emergencies.

### The role of social support on anxiety

Social support serves as a pivotal social resource that individuals leverage, stemming from networks such as friends, family, and significant others (8). Extensive empirical investigations have consistently evidenced that social support is effective in alleviating negative emotional states, including depression, anxiety, and stress (9–12). Moreover, social support has been shown to positively predict emotional well-being, a sense of belonging, and overall individual flourishing (13, 14). Researchers have further examined the intricate relationship between social support and psychological resilience (13, 15). It is plausible that social support can enhance an individual’s psychological resilience, thereby promoting positive mental health outcomes.

Recently, researchers have been interested in how the relationship between social support and mental health is particularly during the COVID-19 pandemic (16–19). For example, a recently study showed that perceived social support has a positive impact on resilience and academic self-efficacy. Additionally, social support, resilience, and academic self-efficacy collectively have a negative influence on the uncertainty associated with COVID-19 (18). Though numerous investigations consistently highlight the protective role of social support in helping individuals manage anxiety (20, 21), critical questions regarding the strength of the association between social support and anxiety, as well as the moderating factors influencing this relationship, remain unanswered.

### Role of moderator variables

In this study, we proposed that the relationship between social support and anxiety is potentially moderated by a constellation of variables, including but not limited to the demographic characteristics of the individuals (such as age and gender), the population involved in the study (identifying the target audience), the phrase of the pandemic under examination, and the encompassing cultural milieu within which the support is exchanged. The goal was to elucidate the nuanced and context-dependent nature of the relationship between social support and anxiety, acknowledging the diverse ways in which these constructs interact in different populations and at varying stages of the major public emergencies.

#### Cultural area

The Inglehart-Welzel cultural map, an instrument extracted from the World Values Survey, is a widely recognized tool that categorizes nations into eight distinct clusters based on their underlying social and cultural value orientations. This map provides a comprehensive framework for understanding the variations in social and cultural values across different nations (22, 23). The clusters identified by the Inglehart-Welzel cultural map represent diverse regions with distinct cultural identities and value systems. For example, cultures in the English-speaking cluster tend to highly value personal independence, individualism, and personal freedom (24). On the other hand, Confucian Cultural Areas prioritize interdependence, collectivism, and social harmony (25). These fundamental value differences have significant implications for how social support is understood and utilized within different cultural contexts. Research has consistently shown that social support plays a critical role in buffering the negative effects of stress and anxiety (9–12). However, the perception and effectiveness of social support can be deeply influenced by cultural norms and expectations. In collectivist cultures, such as those found in Confucian Cultural Areas, social support may be more about maintaining group harmony and less about individual distress (26). In contrast, individualistic cultures may emphasize the importance of personal autonomy and emotional self-regulation when dealing with anxiety. Therefore, this study incorporates cultural area as a moderating variable to examine how the link between social support and anxiety may vary across different cultural contexts.

#### Pandemic phase

Psychological health and social support services have been affected in different ways by different phases of the COVID-19 pandemic (27–29). There has been variation in anxiety symptoms in other stages of lockdown activities. For example, the initial phase of strict lockdowns may have led to a perceived decrease in available social support, resulting in increased anxiety due to the isolation and uncertainty (30). Therefore, we examined whether the connection between anxiety and social support changes throughout the phrase of the pandemic.

### Target audience

It has been found that the different population face varying mental health challenges and experience different levels of social support, based on their unique attributes and exposure to the pandemic (20, 21). For example, during the COVID-19 pandemic, the healthcare workers, especially the environmental services workers, may face greater psychological stress. This stress stems from the high-risk work environment, the intense nature of the work itself, and the multifaceted pressures of interpersonal relationships (31, 32). Given their increased risks and demands, it is likely that the relationship between anxiety and social support among this group is different from that in other populations. Thus, this study considers the target audience as a moderating variable to explore its influence on the relationship between anxiety and social support.

#### Age and gender

Previous studies showed that perceptions of support and mental health outcomes based on age during the COVID-19 pandemic (33–35). Choi et al. (36) found that enhanced social support, including emotional/informational support and positive social interactions, was associated with a lower risk of depression, with age served as significant modifiers of this association. Therefore, age is used as a moderator to examine its role in explaining the relationship between social support and anxiety. In addition, it suggested that the COVID-19 pandemic may exacerbate gender disparities in mental health outcomes (37–39). Some studies have reported more pronounced mental health issues among females, including higher levels of anxiety, depression, and stress (37, 38). However, not all studies have found statistical differences between genders in terms of mental health responses to the pandemic (39). These inconsistent results may be attributed to the complexity of gender as a social construct and the multifaceted nature of mental health outcomes. Thus, this study takes into account the potential moderating effect of gender on the relationship between social support and anxiety.

### The current study

To address the issue of heterogeneity in previous research findings, we employed the meta-analysis method to comprehensively examine the relationship between social support and anxiety during the COVID-19 pandemic. This study used the meta-analytic method and incorporated a substantial sample size (*N* = 107,660), which allows for the identification of patterns and trends across various studies, enhancing the robustness and generalizability of the findings. Furthermore, the analysis incorporates moderating variables, enabling a discussion on the underlying mechanisms governing the relationship between social support and anxiety. By exploring these moderating factors, this study provides valuable insights into the heterogeneity of the social support-anxiety linkage, allowing for a more nuanced understanding of the pathways through which social support can mitigate anxiety.

## Methods

### Ethical Statement

This study, given its non-involvement of human participants, negated the need for informed consent. Ethical approval was, however, diligently obtained from the ethnic committee at Jiangsu Normal University.

### Preregistration statement

No preregistration was conducted in this study.

### Search procedures

A thorough synthesis of existing literature was meticulously conducted to gather all pertinent evidence related to the research topic. The study adopted a systematic exploration of diverse English databases, including Google Scholar, Web of Science, PubMed, PsycINFO, JSTOR, Science Direct, Springer Link, Wiley, Ebscohost, ProQuest, and the Chinese database CNKI. In addition, we have access to three primary types of gray literature through academic libraries, which include theses/dissertations, annual reports, and catalogues.

**Figure 1:**
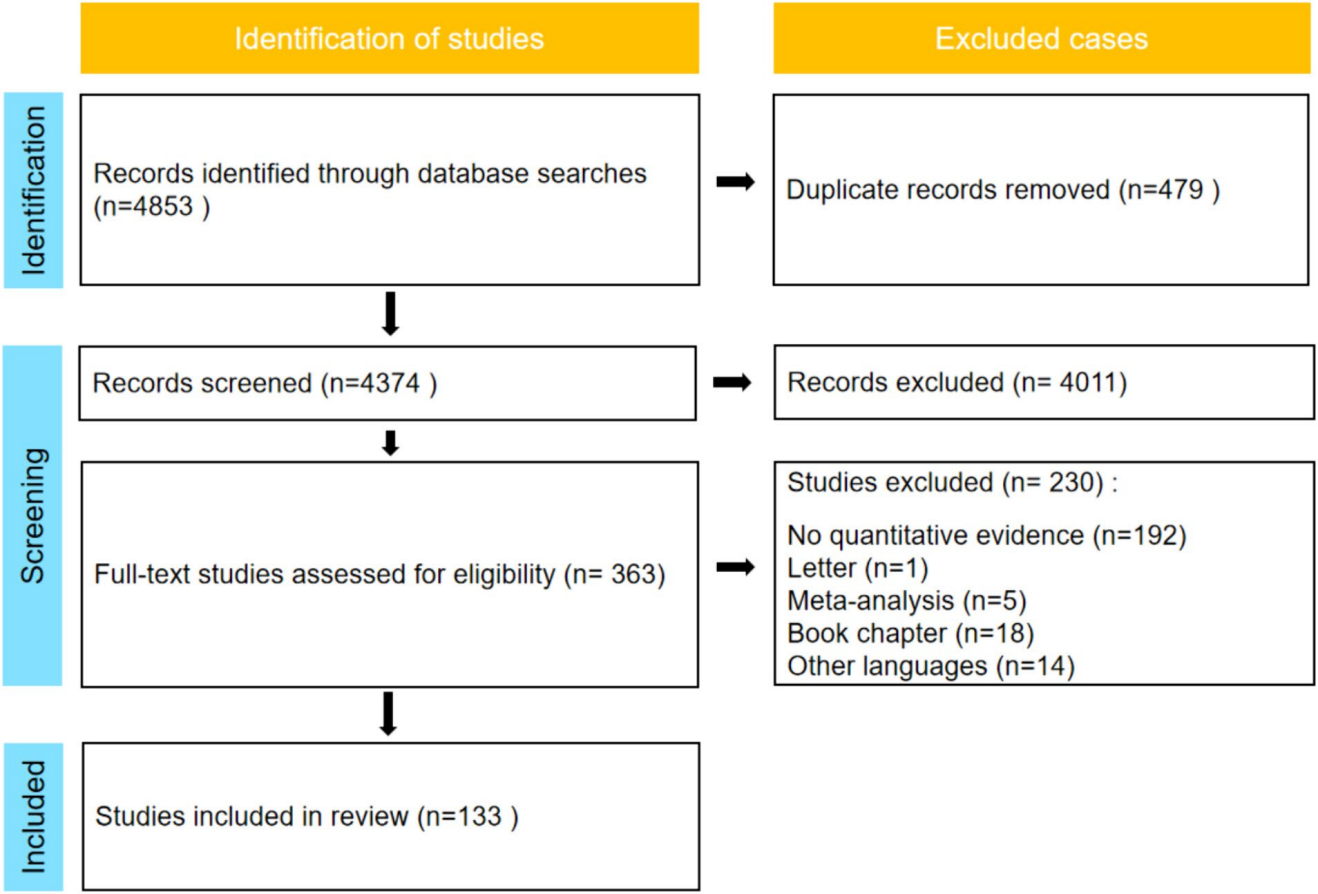
Flowchart of the search and screening process.

The search was confined to articles published up to June 30, 2023. Article titles, keywords, and abstracts were retrieved using a combination of search terms such as ‘COVID-19,’ ‘Coronavirus,’ ‘2019-ncov,’ alongside ‘stress,’ ‘anxiety,’ and ‘social support.’ Additionally, a comprehensive manual reference search was performed on the reference lists of eligible studies, including review studies and meta-analyses identified during the initial search. This approach ensured the inclusion of a diverse array of both published and unpublished works, mitigating the risk of inadvertent oversights in the search process. Figure 1 visually outlines the steps involved in the literature screening process.

### Inclusion criteria

The criteria for selecting literature encompassed the following elements: (1) studies considered for inclusion must have been conducted within the framework of the COVID-19 pandemic; (2) the studies had to employ questionnaires as the designated measurement method; (3) the reported findings needed to include bivariate correlation coefficients between anxiety and social support variables, accompanied by information on the total sample size of participants; (4) literature composed in either English or Chinese was eligible for inclusion.

### Exclusion criteria

The criteria for excluding literature covered several aspects: (1) literature written in languages other than English and Chinese was excluded; (2) meta-analytic or review-type studies were not considered for inclusion; (3) studies that provided separate data for distinct subgroups (e.g., males and females, physicians and non-physicians) instead of reporting correlation coefficients for variables in the overall sample were excluded; (4) studies lacking reported sample sizes were also excluded.

### Literature quality assessment

In this study, we conducted an assessment of the literature’s quality utilizing the JBI Critical Appraisal Tools (40) across eight distinct dimensions: inclusion criteria, sample description, measurement, metrics, identification of confounders, response to confounders, outcome measures, and statistical analyses. Through this rigorous evaluation process, we derived comprehensive quality scores for each literature item (detailed results in Appendix 1). For the purpose of our analysis, we excluded studies that received a quality score of less than 4 (a total of 6 studies) and also disregarded articles published in non-peer-reviewed journals (a total of 23 articles). As a result, our final dataset comprised a total of 104 articles (*N* = 107,660) that met the criteria for inclusion in the analysis.

### Coding

The literature included in the meta-analysis was coded for characteristics, and during the coding process, each article was coded by two researchers for the following information in the literature according to a fixed coding pattern: (a) descriptive information (e.g., title of the literature, year of publication, and information about the authors); (b) sample information (e.g., sample size, number of males, number of females, age, country, cultural area, and target audience); (c) correlation coefficients; and (d) phrase of pandemic. To ensure consistency, one researcher carried out the coding for all the literature, while the other researcher randomly selected two-thirds of the literature for coding, resulting in a concordance rate of 90% or higher. A partial coding list of the literature information is displayed in Table 1.

**Table 1:** Literature information coding table (partial).

Article	Publication status	Pandemic phase	Cultual area	Target audience	Age	*N*	*r*
Abdoli et al. (47)	Published	The second half of 2020	African-Islamic Cultural Area	Health workers	36.86	321	−0.4
Ao et al. (48)	Published	The first half of 2020	Confucian Cultural Area	Common people	NR	736	−0.265
Barros and Sacau-Fontenla (49)	Published	The first half of 2021	Catholic Europe Cultural Area	College students	20.66	923	−0.268
Chen et al. (50)	Published	The first half of 2020	Confucian Cultural Area	Common people	29.28	1921	−0.3
Chinawa et al. (51)	Published		African-Islamic Cultural Area	Primary and secondary school students	16.5	496	−0.195
Costa et al. (52)	Published	The first half of 2020	Catholic Europe Cultural Area	Common people	23.91	1,344	−0.05
Ekmen et al. (53)	Published		African-Islamic Cultural Area	Others	NR	628	0.0094
Grumi et al. (54)	Published		Catholic Europe Cultural Area	maternal	39.72	281	−0.21
Hou et al. (55)	Published	The first half of 2021	Confucian Cultural Area	Health workers	NR	701	−0.391
Muyor-Rodríguez et al. (56)	Published	The first half of 2021	Catholic Europe Cultural Area	College students	21.03	517	−0.095

“NR”, Not reported or cannot be encoded exactly.

The process of study coding adhered to the following principles: (1) Effect values were extracted based on independent samples, ensuring that each independent sample contributed only one effect value; (2) In the process of coding the cultural areas, we referred to the most recent edition of the Inglehart-Welzel cultural map of the world (2020 edition). This classification system grouped the diverse countries into eight distinct clusters, denoted as the English-speaking Cultural Area, Latin American Cultural Area, Orthodox Europe Cultural Area, Catholic Europe Cultural Area, Protestant Europe Cultural Area, African-Islamic Cultural Area, West and South Asia Cultural Area, and Confucian Cultural Area (22); (3) In situations where multiple dimensions of one or more variables were involved, if the overall correlation coefficient between the variables was not reported in the literature, a formula was employed to combine the correlation coefficients, following the approach proposed by Raudenbush (41). The specific formula employed was as follows.


$$r_{xy}=\frac{\sum r_{\mathrm{xi}}r_{yi}}{\sqrt{n+n\left(n-1\right){\bar{r}}_{\mathrm{xixj}}}\sqrt{m+m\left(m-1\right){\bar{r}}_{\mathrm{yiyj}}}}$$

## Results

### Analysis of publication bias

**Figure 2:**
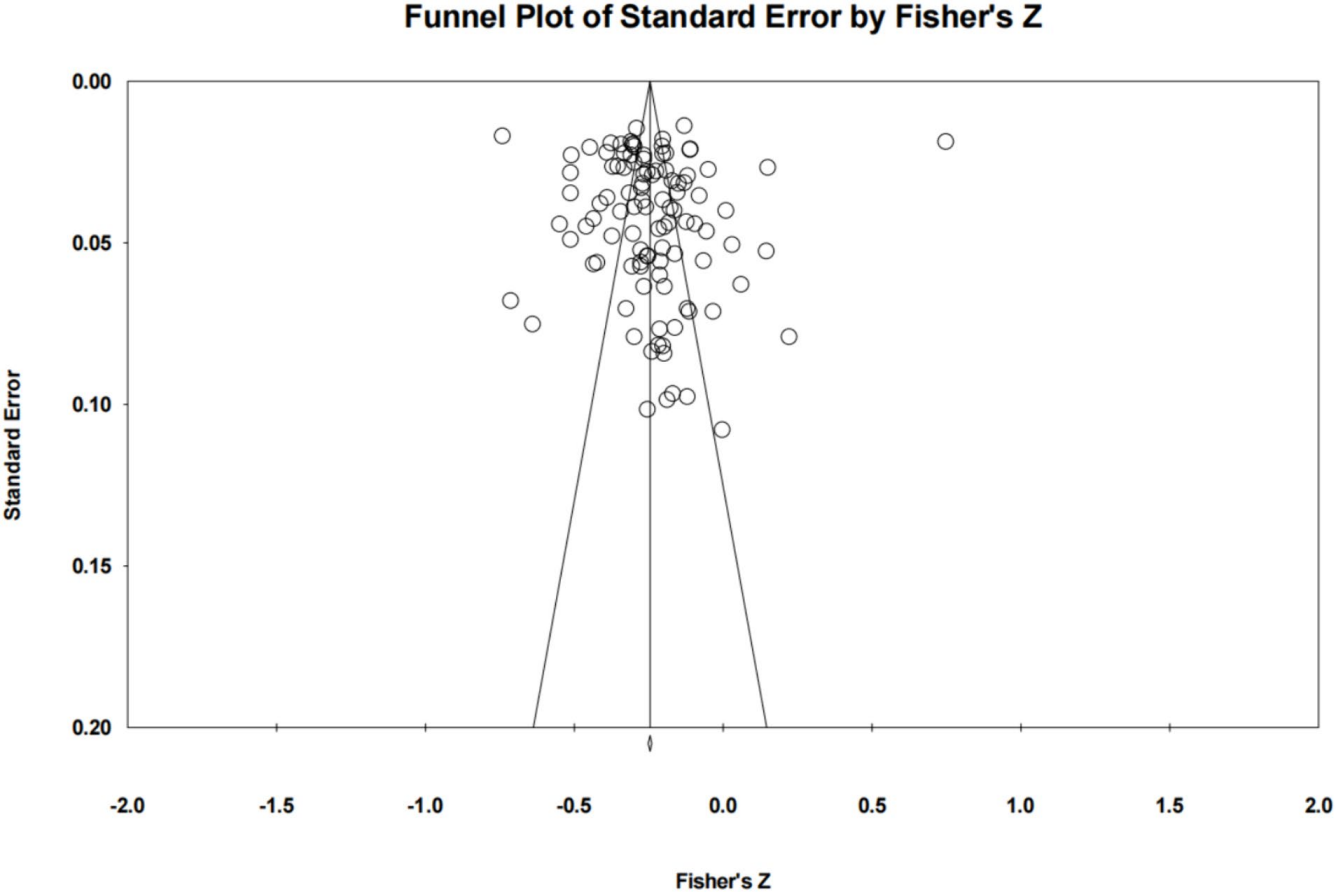
Publication bias funnel plot.

Given the potential impact of publication bias on the integrity of study findings (41), it was imperative to evaluate the presence of such bias within the included literature as a prerequisite for ensuring the reliability of the study outcomes. The assessment of publication bias in this investigation primarily relied on the outcomes of funnel plots (42), along with the fail-safe-N (43) and Egger’s regression tests (44). The meta-analysis encompassed studies that were meticulously examined for any signs of publication bias via the funnel plots depicted in Figure 2. Upon reviewing Figure 2, it is evident that the effect sizes are predominantly clustered above the funnel plot, and they are uniformly distributed on either side of the graph around the aggregate effect size, suggesting a symmetrical pattern.

In order to ensure the absence of publication bias, Rosenthal’s fail-safe number (FSN) value was calculated. The results are provided in Table 2. As seen in Table 2, the FSN was calculated as *N* = 7,704. According to Rosenthal, a high N number will increase the validity of the results obtained with the meta-analysis (45). Moreover, this value is well above the N/5 k + 10 (N: Number of Error Protection; k: Number of studies included in the meta-analysis) limit and is too high to reach (46). This information was accepted as another indication that there was no publication bias and that the results of the meta-analysis were reliable (57). Additionally, the results obtained from the Egger linear regression analysis indicate non-significance, with an intercept of 1.603, 95% CI [−2.800, 3.559]. Consequently, these findings provide substantial evidence to conclude that there is no observable presence of publication bias.

**Table 2:** Rosenthal’s Fail-safe number calculations.

Z-value for observed studies	−71.34582
*p*-value for observed studies	0.00000
Alpha	0.05000
Tails	2
Z for alpha	1.95996
Number of observed studies	104
Fail-safe N	7,704

### Testing for heterogeneity and selection of models

In this study, the Q significance test and I2 index values were utilized to evaluate heterogeneity. If substantial heterogeneity was observed, the random effects model was employed. Otherwise, the fixed effects model was applied (58). The heterogeneity test results indicated a significant level of heterogeneity, with Q-significance values below 0.001 and I2 values exceeding 75% (refer to Table 3). Consequently, considering these findings, the weighted correlations were calculated using the random effects model for this study.

**Table 3:** Results of heterogeneity test and publication bias.

**Relationship**	***K***	***N***	***R***	**95% confidence interval**		***Q*-value**		**Tau-squared**	**Fails *K***	**The regression intercept of Egger**	
				**Lower limit**	**Upper limit**	***Q***	***I*** ^***2***^	***T*** ^***2***^		***Coef.***	***P***
Anxiety-Social support	104	107,660	−0.233	−0.275	−0.191	5380.455^***^	98.086	0.050	137,704	0.379	0.895

***p < 0.05.

### The relationship between social support and anxiety

**Figure 3:**
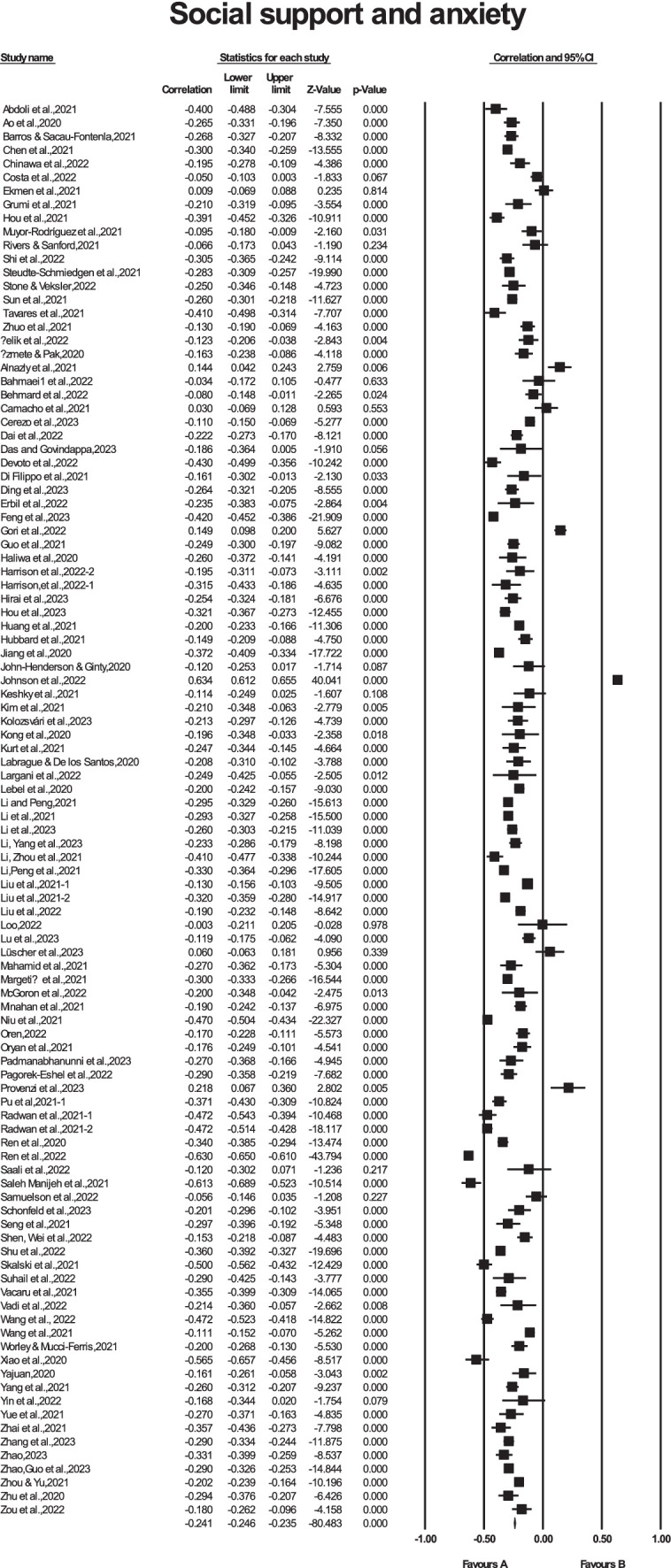
Forest plot of the relationship between social support and anxiety correlation.

The outcomes derived from the random effects model revealed a significant correlation of −0.233 (*K* = 104, 95% CI [−0.275, −0.191]) between social support and anxiety, indicating the presence of a weak negative association between these variables. Figure 3 presents the detailed forest plot illustrating these results.

### Subgroup analysis

The potential moderating effects of various factors on the association between social support and anxiety were thoroughly examined. The results from the moderating effect analysis revealed that age (*Q* = 4.080, *p* = 0.395), sex ratio (Coefficient = 0.0012, *Z* = −0.57, *p* = 0.567), and target audience (*Q* = 12.856, *p* = 0.169) did not exhibit any significant moderating effects on the relationship between social support and anxiety. However, cultural area, and pandemic phase demonstrated a notable moderating effect on the relationship between social support and anxiety (refer to Table 4).

**Table 4:** Analysis of the effects of relevant moderator variables.

Class variable	Level	*K*	*R*	95% confidence interval		*Q*
				Lower limit	Upper limit	
Cultural area	African-Islamic Cultural Area	19	−0.230	−0.321	−0.134	14.120^*^
	Confucian Cultural Area	44	−0.295	−0.334	−0.254	
	Catholic Europe Cultural Area	13	−0.167	−0.276	−0.053	
	West & South Asia Cultural Area	4	−0.181	−0.244	−0.116	
	Protestant Europe Cultural Area	3	0.029	−0.599	0.635	
	English speaking Cultural Area	14	−0.206	−0.261	−0.151	
Pandemic Phase	The first half of 2020	56	−0.220	−0.285	−0.153	13.678^*^
	The second half of 2020	10	−0.258	−0.361	−0.148	
	The first half of 2021	6	−0.275	−0.396	−0.145	
	The second half of 2021	4	−0.235	−0.364	−0.097	
	The first half of 2022	5	−0.316	−0.337	−0.294	
	The second half of 2022	2	−0.224	−0.295	−0.151	

*p < 0.001.

#### Cultural area

In the Confucian Cultural Area, the correlation between social support and anxiety displayed the strongest association with a coefficient of −0.295, significantly higher than correlations observed in other cultural areas. Following closely was the African-Islamic Cultural Area, showing the second-strongest correlation at −0.230. In contrast, the Protestant Europe Cultural Area exhibited a notably weak positive association with a correlation of 0.029, significantly lower than correlations in other cultural areas. Subgroup analysis results indicated a significant difference in group effect sizes (*Q* = 14.120, *p* < 0.05), suggesting varying strengths of the correlation between social support and anxiety across different cultural areas.

#### Pandemic phase

The correlation analysis revealed that the association between social support and anxiety manifested most robustly during the first half of 2022, yielding a weighted correlation coefficient of −0.316. The second-strongest correlation occurred in the first half of 2021, with a coefficient of −0.275. Notably, the weakest correlation was observed in the first half of 2020, with a coefficient of −0.220. Subgroup analysis results indicated a significant difference in group effect sizes (*Q* = 13.678, *p* < 0.05), suggesting varying strengths of the correlation between social support and anxiety across different phrase of the pandemic. Table 4 analysis of the effects of relevant moderator variables area African-Islamic Cultural Area 19–0.230 -0.321 -0.134 14.120* Confucian Cultural.

## Discussion

Numerous researchers have investigated the correlation between social support and public mental health since the onset of the COVID-19 pandemic. Our findings indicate a negative correlation between social support and anxiety, aligning with the outcomes of most previous studies (59, 60). The current meta-analysis presented here provided a comprehensive summary of the existing literature on the relationship between social support and mental health during the COVID-19 pandemic. By analyzing a substantial sample size, our findings contribute to the robustness of the evidence base regarding the role of social support in mitigating the mental health consequences of the COVID-19 pandemic.

We believe that social support has likely played a role in reducing anxiety during the COVID-19 pandemic in at least two aspects. Firstly, anxiety during the pandemic often stems from profound uncertainty [e.g., (61)]. Given the virus’s ongoing novelty and propensity for mutation, individuals are frequently enveloped in a climate of unpredictability, which unequivocally heightens levels of anxiety among the public. However, a recently study indicated that social support can significantly reduce individuals’ uncertainty of COVID-19 [e.g., (18)]. Therefore, amidst the pandemic, the availability of social support has the potential to offer individuals the psychological comfort they require by mitigating uncertainty, thereby resulting in a notable reduction of their anxiety levels. Secondly, the existing research has pointed out that resilience can effectively reduce individuals’ anxiety levels during the COVID-19 pandemic (62). The relationship between social support and resilience is intricately intertwined (63, 64). Social support, in its various forms, serves as a buffer against stress and anxiety, providing individuals with the emotional, informational, and instrumental resources necessary to cope effectively. This support fosters a sense of belonging, self-esteem, and efficacy, which in turn bolsters resilience, enabling individuals to adapt to challenges and adversity with greater flexibility and strength.

The Secondary goal of this current study was to investigate which factors moderate the relationship between social support and the public’s negative mental outcomes, particularly anxiety, in the context of the COVID-19 pandemic. The results from the analysis of moderating effects revealed that cultural context significantly impacted the relationship between social support and anxiety. Notably, the Confucian cultural area exhibited the strongest correlation between social support and anxiety, while the Protestant European cultural area demonstrated the weakest correlation among participants. Collectivist cultural qualities, emphasizing group social cohesion, adherence to social norms, and emotional responsiveness, are typically prevalent in countries within the Confucian Cultural Area, such as, China (65). The collectivist culture can facilitate a more proactive approach to seeking social support, as individuals in such cultures may perceive support-seeking as a normative and necessary response to hardship. This proactive social support-seeking is fostered by a collectivist cultural norm, which in turn can lead to the development of a more extensive social network and a heightened sense of community, serving as crucial resources during major public emergencies. This cultural predisposition likely accounts for the stronger correlation between social support and anxiety observed in the Confucian cultural area. In contrast, the Protestant Europe cultural area, exhibited the weakest correlation among participants. This may be attributed to the cultural values of individualism, autonomy, and egalitarianism that are prevalent in these societies (66). The emphasis on personal freedom and self-determination may lead individuals to rely less on social support and feel more empowered to manage their own anxiety (67). This cultural inclination could explain the weaker correlation between social support and anxiety found in the Protestant European cultural area.

The pandemic phrase has been identified as a critical factor that moderated the relationship between social support and anxiety, with the strength of this association varying depending on the timeframe under consideration. A significant finding from the research is that the first half of 2022 exhibited the strongest correlation between social support and anxiety. It is possible because the phrase saw the benefits of vaccination preventive measures becoming more pronounced, as more individuals were vaccinated and experiencing a sense of security against the virus (68). The gradual relaxation of national lockdown regulations also played a role, as people adjusted to new social norms and experienced a sense of returning to a more normalized way of life, potentially increasing the reliance on social support to navigate these changes (69). In contrast, the first half of 2020, which encompassed the early stages of the pandemic when the situation was particularly dire and stringent lockdown restrictions were implemented, revealed a weaker relationship between social support and anxiety. This weakened correlation can be understood in the context of the immense challenges faced by individuals in accessing social support during this time. The restrictions not only limited physical interactions but also disrupted the normal social networks and support systems (70). The psychological impact of such restrictions, coupled with the fear and uncertainty surrounding the pandemic, likely mitigated the availability of social support, thus weakening the association between social support and anxiety.

In conclusion, social support is a powerful tool in reducing public anxiety during the COVID-19 pandemic. In the future major public emergencies, strategies to enhance social support should be tailored to the specific needs and contexts of different communities. For example, in areas with high levels of social isolation, initiatives to promote digital connectivity and virtual social gatherings can be implemented. In communities with vulnerable populations, such as the older adult or those with chronic illnesses, targeted social support programs, including home visits, telephone check-ins, and mental health counseling, can be provided. In addition, in leveraging social support to alleviate anxiety, it’s imperative to consider both cultural factors and the varying impacts of different pandemic phrase. For instance, in a collectivist culture during the first half of 2022, when vaccination measures were increasingly effective, promoting community-based support groups could be highly effective, as individuals felt safer engaging in group activities. However, in the same culture during the initial lockdown phase in 2020, the same approach may have been less effective due to the stringent restrictions on social gatherings. Conversely, in an individualistic culture, during the same periods, online counseling or one-on-one support sessions tailored to individual needs may be more appropriate. Understanding these nuances allows us to tailor social support strategies to be culturally sensitive and responsive to the changing pandemic landscape.

### Limitation and future directions

In summary, the research presented here has endeavored to capture the multifaceted nature of social support and its relationship with anxiety amid the COVID-19 pandemic. The study underscores the function of social support in bolstering mental well-being within the individuals, acknowledging the nuanced ways in which this support can operate under various underlying conditions. However, it is essential to recognize the limitations of this review to appreciate the scope and implications of the findings. One key limitation is the restrictive inclusion criteria that focused primarily on literature published in Chinese and English. Such a constraint may have inadvertently introduced linguistic bias, potentially overlooking valuable insights from studies conducted in other languages. To address this, future research should strive for inclusivity by encompassing a broader range of linguistic and cultural contexts. Furthermore, it highlights the need to consider the influence of positive psychological attributes, such as resilience and hope, on mental health outcomes during the pandemic. These attributes can serve as protective factors against anxiety and other mental health challenges, thereby modulating the impact of social support. Future studies might explore the interplay between these attributes and the effectiveness of social support mechanisms in buffering against the stresses of pandemics. Finally, considering the ongoing developments of the pandemic and its psychological effects [e.g., (71)], longitudinal studies may yield valuable insights into how the connection between social support and anxiety evolves over time.

## Data availability statement

The raw data supporting the conclusions of this article will be made available by the authors, without undue reservation.

## Ethics statement

The studies involving humans were approved by the ethnical committee at Jiangsu Normal University. The studies were conducted in accordance with the local legislation and institutional requirements. The human samples used in this study were acquired from this is a meta-analysis article. The statistical values were acquired from previous published articles based on literature review. Written informed consent for participation was not required from the participants or the participants' legal guardians/next of kin in accordance with the national legislation and institutional requirements.

## Author contributions

JL: Conceptualization, Formal analysis, Methodology, Writing – review & editing, Visualization. SC: Data curation, Formal analysis, Methodology, Visualization, Writing – original draft. ZW: Conceptualization, Writing – review & editing, Data curation, Formal analysis, Funding acquisition, Investigation, Methodology, Supervision, Writing – original draft. FR: Conceptualization, Writing – review & editing.
